# Construction and Analysis of a ceRNA Network in Cardiac Fibroblast During Fibrosis Based on *in vivo* and *in vitro* Data

**DOI:** 10.3389/fgene.2020.503256

**Published:** 2021-01-21

**Authors:** Qing-Yuan Gao, Hai-Feng Zhang, Zhi-Teng Chen, Yue-Wei Li, Shao-Hua Wang, Zhu-Zhi Wen, Yong Xie, Jing-Ting Mai, Jing-Feng Wang, Yang-Xin Chen

**Affiliations:** ^1^Department of Cardiology, Sun Yat-sen Memorial Hospital, Sun Yat-sen University, Guangzhou, China; ^2^Laboratory of Cardiac Electrophysiology and Arrhythmia in Guangdong Province, Guangzhou, China

**Keywords:** cardiac fibroblast, fibrosis, competing endogenous RNA, ADAM metallopeptidase domain 19, transforming growth factor beta induced

## Abstract

**Aims:**

Activation of cardiac fibroblasts (CF) is crucial to cardiac fibrosis. We constructed a cardiac fibroblast-related competing endogenous RNA (ceRNA) network. Potential functions related to fibrosis of “hub genes” in this ceRNA network were explored.

**Materials and Methods:**

The Gene Expression Omnibus database was searched for eligible datasets. Differentially expressed messenger (m)RNA (DE-mRNA) and long non-coding (lnc)RNA (DE-lncRNA) were identified. microRNA was predicted and validated. A predicted ceRNA network was constructed and visualized by Cytoscape, and ceRNA crosstalk was validated. A Single Gene Set Enrichment Analysis (SGSEA) was done, and the Comparative Toxicogenomics Database (CTD) was employed to analyze the most closely associated pathways and diseases of DE-mRNA in the ceRNA network. The functions of DE-mRNA and DE-lncRNA in the ceRNA network were validated by small interfering (si)RNA depletion.

**Results:**

The GSE97358 and GSE116250 datasets (which described differentially expressed genes in human cardiac fibroblasts and failing ventricles, respectively) were used for analyses. Four-hundred-and-twenty DE-mRNA and 39 DE-lncRNA, and 369 DE-mRNA and 93 DE-lncRNA were identified, respectively, in the GSE97358 and GSE116250 datasets. Most of the genes were related to signal transduction, cytokine activity, and cell proliferation. Thirteen DE-mRNA with the same expression tendency were overlapped in the two datasets. Twenty-three candidate microRNAs were predicted and the expression of 11 were different. Only two DE-lncRNA were paired to any one of 11 microRNA. Finally, two mRNA [ADAM metallopeptidase domain 19, (*ADAM19*) and transforming growth factor beta induced, (*TGFBI*)], three microRNA (*miR-9-5p*, *miR-124-3p*, and *miR-153-3p*) and two lncRNA (*LINC00511* and *SNHG15*) constituted our ceRNA network. siRNA against *LINC00511* increased *miR-124-3p* and *miR-9-5p* expression, and decreased *ADAM19* and *TGFBI* expression, whereas siRNA against *SNHG15* increased *miR-153-3p* and decreased *ADAM19* expression. *ADAM19* and *TGFBI* were closely related to the TGF-β1 pathway and cardiac fibrosis, as shown by SGSEA and CTD, respectively. Depletion of two mRNA or two lncRNA could alleviate CF activation.

**Conclusions:**

The CF-specific ceRNA network, including two lncRNA, three miRNA, and two mRNA, played a crucial role during cardiac fibrosis, which provided potential target genes in this field.

## Introduction

Cardiac fibrosis, a pivotal part of cardiac remodeling and heart failure, is a considerable global health problem that derives from many forms of heart diseases all over the world (Kong et al., 2014; Rockey et al., 2015; Ziaeian and Fonarow, 2016; Park et al., 2019). Cardiac fibrosis resulting from increased deposition of the extracellular matrix (ECM) is caused by enhanced cardiac fibroblasts (CF) activity. The latter are important for sustaining ECM homeostasis, and are essential cells for the normal structure and function of the heart. However, under certain types of stress or injury (e.g., hypoxia, pressure/volume overload), the heart produces pro-fibrotic factors, such as transforming growth factor (TGF)-β1, angiotensin II (Ang-II), and platelet-derived growth factor (PDGF), which can transform CF into myofibroblasts. This action results in CF activation, and is the key event in the development of cardiac fibrosis (Travers et al., 2016).

Cardiac fibrosis is considered to be the outcome of molecular modulations. Besides coding RNA [which is mainly messenger (m)RNA], the role of non-coding RNA (ncRNA) in heart disease has garnered scientific interest, and the ncRNA transcriptome is altered substantially in cardiac fibrosis (Creemers and van Rooij, 2016; Poller et al., 2018; Hao et al., 2019; Hobuß et al., 2019; Zheng et al., 2019; Luo et al., 2020). Among all the ncRNA, microRNA and long non-coding RNA (lncRNA) are investigated the most and are believed to be a vitally important component in this kind of RNA. Among all the interactions between mRNA and ncRNA, competing endogenous RNA (ceRNA) is a critical interaction (Huang et al., 2017). ceRNA is a gene regulator that is present in physiologic and pathologic conditions (Kartha and Subramanian, 2014; Tay et al., 2014). RNA transcripts such as mRNA, pseudogenes, lncRNA, circular RNA, or other molecules that share the same specific microRNA binding sites can combine with microRNA competitively (Credendino et al., 2019). Thus, the inhibitory effects of gene expression conferred by microRNA are attenuated and cause a cascade of gene actions (Fischer, 2015).

ceRNA can serve as an important regulatory mechanism in several cardiovascular diseases. A ceRNA network, relating to myocardial infarction using human data, has been built, the results of which could improve our understanding of the molecular mechanisms underlying myocardial infarction (Zhang et al., 2018). Studies have shown that ncRNA modulates the progression of cardiac fibrosis, and that ceRNA also plays an important part in it (Liang et al., 2018; Zhao et al., 2018; Sun et al., 2019), but a ceRNA network that shows regulatory actions among coding RNA and ncRNA is not available.

Therefore, we analyzed RNA-sequencing data derived from fibrotic human ventricles and activated human CF. In this way, we predicted a ceRNA regulatory network to further understand the molecular mechanisms of CF during cardiac fibrosis.

## Materials and Methods

### Cell Culture and Small Interfering (si)RNA Transfection

Ventricular human CF (HCF) were obtained from ScienCell Research Laboratory (6310, ScienCell, Carlsbad, CA, United States) and cultured in fibroblast medium-2 containing 5% fetal bovine serum, 1% fibroblast growth supplement-2 (ScienCell), and 1% penicillin and streptomycin (Invitrogen, Carlsbad, CA, United States) at 37°C with an atmosphere of 5% CO_2_. Cells were passaged at 80% confluence, and experiments were carried out using the fifth passage. HCF were treated with classical fibrotic stimuli (human TGF-β1, 10 ng/mL) (R&D Systems, Minneapolis, MN, United States); human Ang-II, 1 μM (Sigma–Aldrich, St. Louis, MO, United States); human PDGF-BB, 10 ng/mL (PeproTech, Rocky Hill, NJ, United States) for 24 h. The same volume of phosphate-buffered saline (PBS) was used for control groups. Further, HCF isolated from atria (HCF-aa) were also obtained from ScienCell Research Laboratory (6320, ScienCell). In the fifth passage, HCF-aa were treated with human TGF-β1 (10 ng/mL, R&D Systems) or PBS for further validation of genes within the ceRNA network.

For siRNA transfections, siRNA [against the target gene or non-sense control (NC)] at 50 nM was transfected into HCF using Lipofectamine^TM^ RNAiMAX (Invitrogen) 24 h before treatments (TGF-β1 or PBS). Two siRNA oligonucleotides targeting one lncRNA were used to avoid off-target effects. All siRNA was provided by IGE (Guangzhou, Guangdong, China). Details of the siRNA sequences are listed in Supplementary Table 1.

### Search for a Candidate Dataset From the Gene Expression Omnibus (GEO) Database

The GEO database is a publicly available genomics data repository containing array- and sequencing-based data. The search terms were “cardiac fibrosis” or “fibrosis,” and only datasets related to cardiac fibrosis were retrieved. Array- and sequencing-based data were considered, as were high-throughput platforms.

### Data Preprocessing, Screening for Differentially Expressed Genes, and Functional Enrichment Analysis

Expression data were normalized by reads per kilobase of transcript per million mapped reads (RPKM), which were then log_2_-transformed for subsequent analyses (Mortazavi et al., 2008). Gene annotation was carried out using the Perl program^1^. The “limma” package (Ritchie et al., 2015) in R v3.6.1 (R Center for Statistical Computing, Vienna, Austria) was used to identify differentially expressed mRNA molecules (hereafter termed “DE-mRNA”) and differentially expressed lncRNA molecules (hereafter termed “DE-lncRNA”) under the criteria of log_2_ fold-change (>1 or <−1) and adjusted *p*-value (<0.05) from the Student’s *t*-test. Heatmaps were produced using the “gplots” (Warnes et al., 2016), “RcolorBrewer” (Neuwirth, 2014), and “ggplot2” (Wickham, 2011) packages in R v3.6.1.

The Gene Ontology (GO) and Kyoto Encyclopedia of Genes and Genomes (KEGG) databases were used for analyses. We employed the Database for Annotation, Visualization and Integrated Discovery (DAVID) algorithm (Huang et al., 2009a,b) to predict the potential functions of DE-mRNA. *p* < 0.05 denoted significant enrichment.

### Construction of a ceRNA Network and ceRNA Validation

DE-mRNA with the same tendency of expression in both datasets were used to predict potentially differentially expressed microRNA (hereafter termed “DE-microRNA”). Five bioinformatics formulae, miRanda (last update: November 01, 2010), Pictar (last update: March 26, 2007), PITA (last update: August 31, 2008), miRmap v1.1, and TargetScan v7.2 (Enright et al., 2003; John et al., 2004; Krek et al., 2005; Chen and Rajewsky, 2006; Kertesz et al., 2007; Vejnar and Zdobnov, 2012; Agarwal et al., 2015) were used to predict the most plausible microRNA. Only microRNA that met the criteria of all five formulae were enrolled.

Potential DE-microRNA expressed during the CF activation identified above were confirmed by reverse transcription-quantitative real time polymerase chain reaction (RT-qPCR). There are few nucleotide differences in some RNA (*miR-30a-5p*/*miR-30d-5p* cluster, *miR-92a-3p/miR-92b-3p* cluster and *miR-106a-5p*/*miR-20a-5p*/*miR-20b-5p*/*miR-17-5p/93-5p* group) and distinguishing the expression profile of each microRNA using RT-qPCR is difficult. Therefore, their RT-qPCR data contained the expression profile of the whole cluster.

ceRNA pairs were considered between microRNA–mRNA binding and microRNA–lncRNA binding. The confirmed DE-microRNA was used directly in construction of the ceRNA network. The binding of lncRNA to microRNA could modulate the interactions between microRNA and target mRNA. Therefore, the StarBase database v3.0 was used to predict more potential lncRNA based on the aforementioned microRNA (Li et al., 2014). Only the predicted lncRNA in the DE-lncRNA list was used for network construction. Expression of the selected genes was validated. A visualized ceRNA network was constructed using Cytoscape v3.7.0 (Shannon et al., 2003). For ceRNA validation, siRNA against two lncRNA, *LINC00511* and *SNHG15*, was transfected, and quantitative dynamic changes in the expression profiles of the microRNA and mRNA in the ceRNA network were detected.

### Functional Analysis of mRNA and lncRNA in the ceRNA Network

The potential functions of mRNA in the ceRNA network were explored by Single Gene Set Enrichment Analysis (SGSEA) and the Comparative Toxicogenomics Database (CTD) initially. SGSEA was conducted to analyze the most closely associated pathways of DE-mRNA in the ceRNA network, as reported previously (Ma et al., 2020). Briefly, median expression of a single mRNA in the ceRNA network was identified among TGFβ1-treated cells *in vitro* or fibrotic ventricles *in vivo*, which were then divided into two groups according to median expression of the target mRNA. Differences in gene-expression profiles in the two groups (expression of target mRNA higher or lower than the median expression) were analyzed using GSEA v4.0.3, and gene sets were enriched to pathways in the KEGG database. Furthermore, we set 1,000 permutations throughout SGSEA (Mootha et al., 2003; Subramanian et al., 2005). CTD was used to explore possible relationships between DE-mRNA in the ceRNA network and disease phenotypes, as reported previously (Davis et al., 2019). After these bioinformatics analyses, the roles of mRNA and lncRNA in the ceRNA network during CF activation were investigated by siRNA experiments.

### Molecular Biological and Biochemical Measurements

RT-qPCR was undertaken to quantify expression of predicted DE-microRNA and all the RNA in the ceRNA network. Briefly, total RNA was extracted using TRIzol Reagent (Invitrogen^®^) and was employed for reverse transcription using the PrimerScript^TM^ RT Regent kit (for microRNA) or PrimerScript^TM^ RT Master kit (for mRNA and lncRNA), both of which were purchased from TaKaRa Biotechnology, (Shiga-ken, Japan). qPCR was carried out with SYBR Reagent (TaKaRa Biotechnology). The primers used are listed in Supplementary Table 2.

Western blotting was undertaken to detect the protein of corresponding DE-mRNA in the ceRNA network and fibrogenesis-associated proteins (actin alpha 2, smooth muscle (*ACTA2*) and collagen type I alpha 1 chain (*COL1A1*). In brief, total proteins were extracted using a commercially available kit (Cell Signaling Technology, Danvers, MA, United States). Proteins were subjected to sodium dodecyl sulfate–polyacrylamide gel electrophoresis, which were then transferred to polyvinylidene difluoride (PVDF) membranes (Millipore, Bedford, MA, United States). PVDF membranes were blocked in 5% bovine serum albumin (Sigma–Aldrich) in Tris-Buffered Saline with 0.1% Tween20 (Sigma–Aldrich) and sequential incubations with primary and secondary antibodies (*ACTA2*) (ab5694, 1:1000, Abcam, Cambridge, United Kingdom), *COL1A1* (ab34710, 1:1000, Abcam), transforming growth factor beta induced (*TGFBI*) (ab170874, 1:1000, Abcam), β*-actin* (4970S, 1:1000, Cell Signaling Technology), connective tissue growth factor (*CTGF*) (MAB91901, 1:500, R&D Technology), ADAM metallopeptidase domain 19 (*ADAM19*) (PA5-84258, 1:1000, Invitrogen) and horseradish peroxidase-conjugated anti-rabbit immunoglobulin G (7074S, 1:1000, Cell Signaling Technology) carried out. Enhanced electrochemiluminescence reagents (Millipore, Billerica, MA, United States) were used to visualize the contents of proteins on PVDF membranes.

To determine collagen production, quantification of *COL1A1* expression in cell-culture supernatants was done using the Human Pro-Collagen I Alpha 1 DuoSet ELISA (R&D Systems). The total secreted collagens were quantified by the Sirius Red Collagen Detection kit (Chondrex, Redmond, WA, United States).

HCF proliferation was measured using the CellTiter 96 AQueous One Solution Cell Proliferation Assay (Promega, Fitchburg, WI, United States) according to the manufacturer’s instructions. In brief, 20 μL of CellTiter 96 AQueous One Solution was added to each well with 100 μL cultured medium in 96 well cell culture plate (Corning, New York, United States), and absorbance at 490 nm recorded 1-h later.

### Statistical Analyses

Data with a normal distribution are presented as the mean ± SEM. One-way ANOVA was used to analyze differences among multiple groups followed by the SNK test for multiple *post hoc* comparisons. The two-tailed student’s *t*-test was used to analyze differences between groups. Statistical analyses were carried out using R v3.6.1. *P* < 0.05 was considered significant.

## Results

### Differentially Expressed Genes During Cardiac-Fibrotic Insult

We searched the GEO database, and enabled retrieval of 344 datasets. However, only two of them (GSE97358 and GSE116250) met the enrollment criteria, so they were used for further analyses. Data in GSE97358 were obtained from CF derived from the right atrium, which then received treatment with TGF-β1 or PBS. One hundred and sixty-eight samples (84 for TGF-β1- and PBS-treated cells, respectively) were included. Data in GSE116250 were obtained from 50 human failing ventricles (37 dilated cardiomyopathies and 13 ischemic cardiomyopathies) and 14 non-failing ventricles from healthy donors. Poly-A enriched RNA-Seq was carried out. Therefore, this dataset contained mRNA and some lncRNA with poly-A. Both of the RNA-sequencing procedures were completed in the HiSeq 2500 platform (Illumina, San Diego, CA, United States).

**FIGURE 1:**
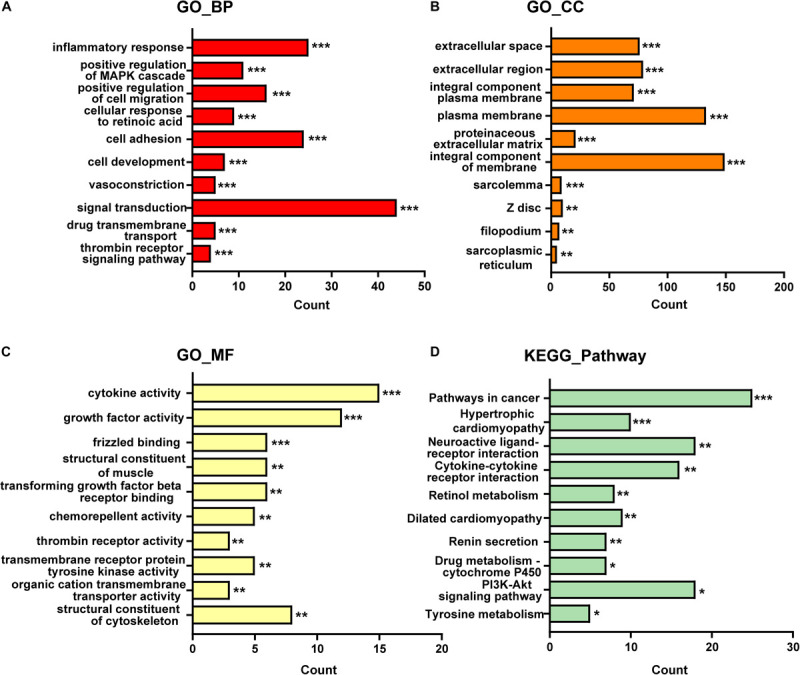
The top 10 items of GO and KEGG enrichment analysis for DE-mRNAs in GSE97358. **(A)** GO-biological processes analysis of top 10 items. **(B)** GO-cellular components analysis of top 10 items. **(C)** GO-molecular functions analysis of top 10 items. **(D)** KEGG pathway analysis of top 10 items. The *x*-axis indicated the counts of enrichment genes. *P < 0.05, **P < 0.01, ***P < 0.001.

A total of 420 DE-mRNA (170 up-regulated and 250 down-regulated) and 34 DE-lncRNA (13 up-regulated and 21 down-regulated) were identified in the GSE97358 dataset. To explore the potential functions of DE-mRNA, GO and KEGG enrichment analysis was used to explore plausible mechanisms and pathways during the progression of cardiac fibrosis. According to the results of the GO analysis, 125 items were identified, 81, 21, and 23 of which were enriched in GO-biological processes (GO-BP), GO-cellular components (GO-CC), and GO-molecular functions (GO-MF), respectively. The top-10 items of GO-analysis results are shown in Figure 1. Briefly, most genes were enriched in signal transduction (GO-BP) (Figure 1A) and cytokine activity (GO-MF) (Figure 1C) pathways. Pathways with the top-10 numbers of enriched genes according to the analysis of the KEGG database were provided in Figure 1D. Pathways in cancer enriched most genes, followed by the *PI3K*-*Akt* signaling pathway. These observations may confirm (as widely accepted) the importance of aging, growth, and proliferation of CF during cardiac fibrosis. Of note, KEGG analysis also displayed that a relatively large number of DE-mRNA was enriched in the interaction between cytokines and cytokine receptors, which supported the results of GO-MF, showing that the largest number of genes was enriched in cytokine activity (Figure 1C).

**FIGURE 2:**
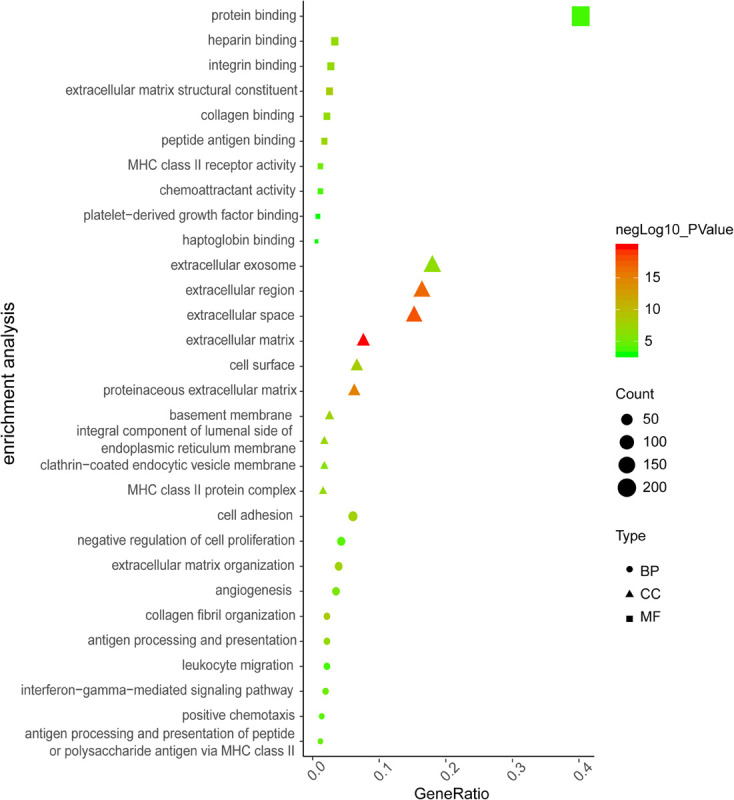
The top 10 items of GO enrichment analysis for DE-mRNAs in GSE116250. The color indicates value of negative log10 (*P*-value), dot size indicates enriched gene counts, and shape indicates the type of GO analysis.

Analysis of GSE116250 (from fibrotic cardiac tissues) identified 369 DE-mRNA (287 up-regulated and 82 down-regulated) and 93 DE-lncRNA (54 up-regulated and 39 down-regulated). The top-10 items of GO-analysis results are provided in Figure 2. Most genes were enriched in the processes of negative regulation of cell proliferation (GO-BP) and calcium-ion binding (GO-MF). Unexpectedly, no classical pathways of cardiac fibrosis were found by analyses of the KEGG database.

**FIGURE 3:**
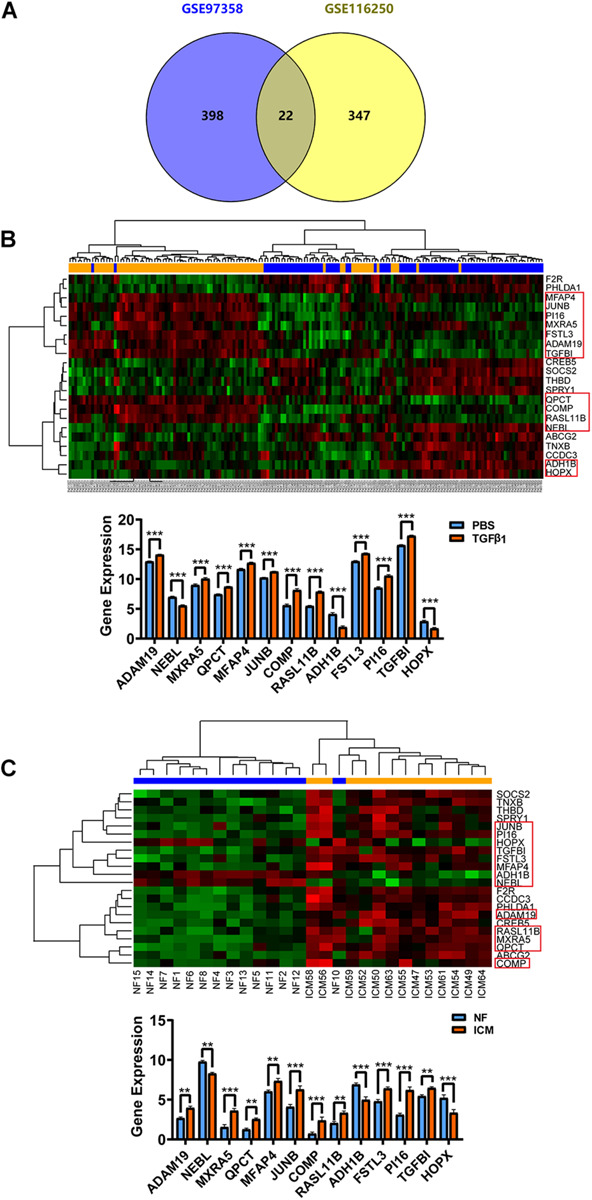
Cardiac fibroblast-specific DE-mRNA derived from the overlap of *in vivo* (GSE116250) and *in vitro* (GSE97358) data. **(A)** Venn diagram for the 22 DE-mRNA overlap in GSE116250 and GSE97358 datasets. **(B)** Heatmap of the 22 DE-mRNA and statistical analysis for relative expression of the 13 DE-mRNA derived from the GSE97358 dataset. **(C)** Heatmap of the 22 DE-mRNA and statistical analysis for relative expression of the 13 DE-mRNA in the GSE116250 dataset. Thirteen DE-mRNA symbolized by a rectangle denoted that the expression tendency of these mRNA was toward the same direction. *P < 0.05, **P < 0.01, ***P < 0.001.

To more visually show the expression of DE-mRNA, based on fold change, the heatmaps for top100 DE-mRNA in GSE97358 and GSE116250 are provided in Supplementary Figures 1, 2, respectively. For expression of DE-lncRNA, the heatmaps for 39 DE-lncRNA in GSE97358 and 93 DE-lncRNA in GSE116250 are shown in Supplementary Figures 3, 4. More important, to yield more plausible differentially expressed genes during cardiac fibrosis, we selected genes identified by both datasets. A total of 22 mRNA in the DE-mRNA lists were identified from GSE97358 and GSE116250 datasets. The genomic parameters and fibrosis-related information of the 22 DE-mRNA are listed in Table 1. It is worth noting that the expression tendencies of 13 genes were changed toward the same direction. The Venn diagram, heatmap, and relative gene expression of these 13 DE-mRNA are presented in Figure 3. Briefly, except for three genes whose expression was down-regulated in the fibrosis group, expression of most of the DE-mRNA in this group was up-regulated.

**TABLE 1:** Twenty-two DE-mRNA genes identified from GSE97358 and GSE116250 datasets.

**Gene name**	**Chromosome location**	**Transcript length (base pair)**	**Score for fibrosis in the Comparative Toxicogenomics database**
Tenascin XB (*TNXB*)	Chr 6: 31, 998, 796-32, 043, 729	3125	58.44
ADAM metallopeptidase domain 19 (*ADAM19*)	Chr 5: 157, 395, 534-157, 575, 775	2823	84.39
Pleckstrin homology like domain family A member 1 (*PHLDA1*)	Chr 12: 76, 025, 447-76, 033, 932	5741	142.55
Nebulette (*NEBL*)	Chr 10: 20, 779, 973-21, 174, 187	9216	72.12
Thrombomodulin (*THBD*)	Chr 20: 23, 045, 633-23, 049, 741	4109	157.58
Matrix remodeling associated 5 (*MXRA5*)	Chr X: 3, 308, 565-3, 346, 652	9804	64.88
Glutaminyl-peptide cyclotransferase (*QPCT*)	Chr 2: 37, 342, 827-37, 373, 322	582	87.48
cAMP responsive element binding protein 5 (*CREB5*)	Chr 7: 28, 299, 321-28, 825, 894	2294	82.74
ATP binding cassette subfamily G member 2 (*ABCG2*)	Chr 4: 88, 090, 150-88, 231, 628	4844	174.35
Microfibrillar associated protein 4 (*MFAP4*)	Chr 17: 19, 383, 442-19, 387, 240	1876	94.03
Coiled-coil domain containing 3 (*CCDC3*)	Chr 10: 12, 896, 625-13, 099, 652	2747	45.16
Sprouty RTK signaling antagonist 1 (*SPRY1*)	Chr 4: 123, 396, 795-123, 403, 760	641	99.85
JunB proto-oncogene, AP-1 transcription factor subunit (*JUNB*)	Chr 19: 12, 791, 486-12, 793, 315	1830	183.24
Cartilage oligomeric matrix protein (*COMP*)	Chr 19: 18, 782, 773-18, 791, 305	2707	66.76
Coagulation factor II thrombin receptor (*F2R*)	Chr 5: 76, 716, 126-76, 735, 770	3727	141.45
RAS-like family 11 member B (*RASL11B*)	Chr 4: 52, 862, 317-52, 866, 835	1968	104.94
Suppressor of cytokine signaling 2 (*SOCS2*)	Chr 12: 93, 569, 814-93, 583, 487	2761	126.91
Alcohol dehydrogenase 1B (class I), beta polypeptide (*ADH1B*)	Chr 4: 99, 304, 971-99, 352, 760	4067	56.58
Follistatin like 3 (*FSTL3*)	Chr 19: 676, 392-683, 392	2501	72.62
Peptidase inhibitor 16 (*PI16*)	Chr 6: 36, 948, 263-36, 964, 837	2288	50.86
Transforming growth factor beta induced (*TGFBI*)	Chr 5: 136, 028, 988-136, 063, 818	2712	158.86
HOP homeobox (*HOPX*)	Chr 4: 56, 647, 988-56, 681, 899	1123	112.52

### Identification of RNA for Construction of the ceRNA Network and ceRNA Validation

A dataset on DE-microRNA between cardiac fibrosis and controls was not available. To obtain DE-microRNA that were essential for construction of the ceRNA network, we predicted microRNA potentially targeting the 13 DE-mRNA identified above. To yield the most plausible microRNA, only those that could be predicted by all five frequently used bioinformatics formulae (miRanda, miRmap, PITA, PicTar and Targetscan) were adopted and, finally, 23 microRNA were retrieved.

**FIGURE 4:**
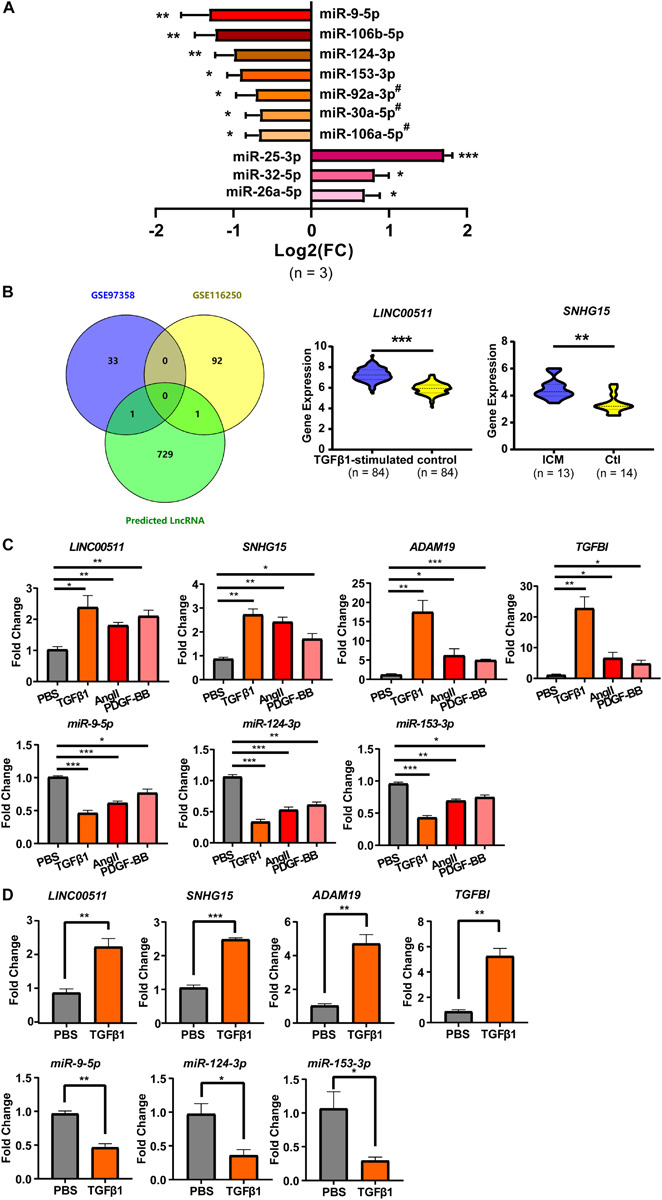
Cardiac fibroblast-specific DE-microRNA and DE-lncRNA. **(A)** RT-qPCR validation of microRNA predicted from the 13 DE-mRNA (shown in Figure 1). *n* = 3 per group. **(B)** DE-lncRNA derived from the overlap of GSE116250 and GSE97358 datasets. **(C)** RT-qPCR validation of selected two mRNA, three microRNA and two lncRNA in TGFβ 1-, AngII- and PDGF-BB-treated HCFs. **(D)** RT-qPCR validation of the two mRNA, three microRNA, and two lncRNA in PBS-treated HCF-aa and TGFβ1-treated HCF-aa. *n* = 3 per group. *P < 0.05, **P < 0.01, ***P < 0.001. #, results represent the microRNA cluster due to few nucleotides difference inside the cluster. *miR-92a-3p*, *miR-92a-3p*/*92b-3p* cluster; *miR-30a-5p*, *miR-30a-5p*/*30d-5p* cluster; *miR-106-5p*, *miR-106a-5p*/*20a-5p/20b-5p/17-5p/93-5p* cluster.

To explore the expression profiles of these microRNA during cardiac fibrosis, we confirmed their expression in HCF obtained from ScienCell Research Laboratory. Summarized results of expression of these microRNA is shown in Figure 4A. Sixteen expression data on microRNA (or microRNA clusters) are presented. All microRNA (or microRNA clusters) except five (*miR-367-3p*, *miR-26b-5p*, *miR-874-3p*, *miR-590-5p*, and *miR-1297*) showed significantly different expression between PBS treatment and TGF-β1 treatment (Figure 4A). Among these, expression of three microRNA (*miR-26a-3p*, *miR-32-5p*, and *miR-25-3p*) was up-regulated by TGF-β1 treatment, whereas expression of eight microRNA (or clusters) was suppressed (Figure 4A). Expression of *miR-25-3p*, *miR-9-5p*, *miR-106a-5p* cluster, *miR-124-3p*, and *miR-153-3p* was down-regulated most significantly (Figure 4A).

The confirmed DE-microRNA was used to predict their interactions with lncRNA, which are also necessary for construction of the ceRNA network. Only the predicted lncRNA that were also included in the DE-lncRNA list were considered for construction of the ceRNA network. The initial prediction retrieved 731 lncRNA, only two of which were included in the DE-lncRNA list (*LINC00511* and *SNHG15*) (Figure 4B). Expression of both was up-regulated to more than twofold under fibrosis. These two lncRNA were shown to interact with three microRNA (*miR-124-3p*, *miR-153-3p*, and *miR-9-5p*), which were shown to target two mRNA (*ADAM19* and *TGFBI*).

To confirm further the reliability of the selected genes for construction of the ceRNA network, besides microRNA expression (which had been validated as described above), expression of mRNA and lncRNA was also confirmed. Results are summarized in Figure 4C. Expression of all the mRNA and lncRNA was up-regulated but expression of 3 microRNA was down-regulated significantly by TGF-β1, Ang-II and PDGF-BB, with the most prominent changes being found upon TGFβ1-treatment. As shown in Figure 4D, we further validated the expression of these genes in additional CF, HCF-aa, which was consistent with findings in HCF.

**FIGURE 5:**
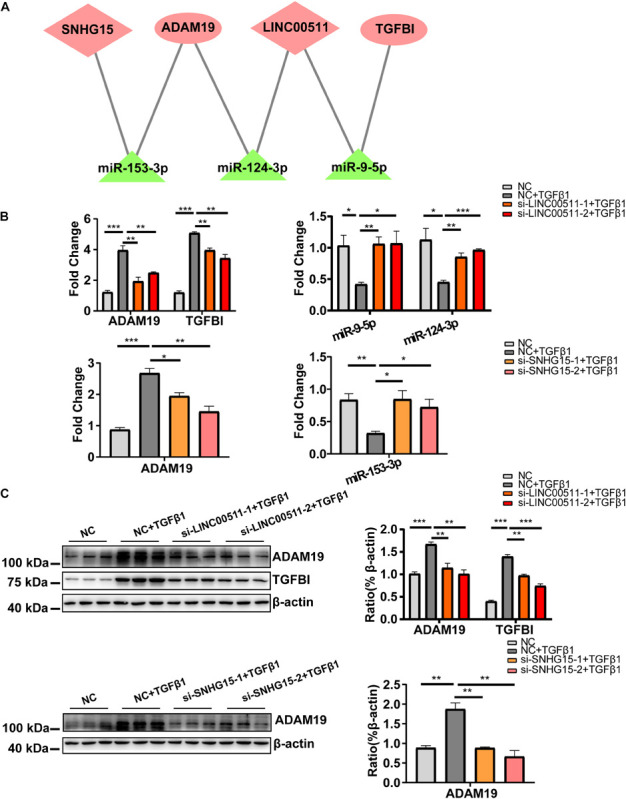
Construction and validation of a cardiac fibrosis ceRNA network. **(A)** ceRNA network of cardiac fibroblast-specific differentially expressed genes during fibrosis. Pink indicates up-regulated genes and green indicates down-regulated genes. **(B)** RT-qPCR validation for quantitative dynamic changes of three microRNA and two mRNA under *LINC00511* and LncRNA *SNHG15* depletion. **(C)** Dynamic changes in protein level of *ADAM19* and *TGFBI*. *n* = 3 per group. *P < 0.05, **P < 0.01, ***P < 0.001.

By prediction using bioinformatics analysis under strict criteria and confirmation of expression profiles, we built the ceRNA network on the basis of microRNA, lncRNA and mRNA. Finally, three microRNA, two lncRNA, and two mRNA were included. The predicted ceRNA network is shown in Figure 5A. As shown, each microRNA targeted one mRNA and one lncRNA. *ADAM19* and *LINC00511* were targeted by two microRNA, whereas *SNHG15* and *TGFBI* were targeted by one microRNA.

We wished to validate the predicted ceRNA network in cardiac fibrosis. Hence, the quantitative dynamic changes of the three microRNA and two mRNA in the network were detected under knockdown of expression of *LINC00511* and *SNHG15* in HCF (the knockdown efficiency of two lncRNA are shown in Supplementary Figure 5). Compared with the TGFβ1-treated group, expression of *miR-9-5p* and *miR-124-3p* was obviously increased, whereas that of *ADAM19* and *TGFBI* was decreased, in cells with siRNA-*LINC00511* transfection (Figure 5B). Similarly, *miR-153-3p* expression was increased and *ADAM19* expression was decreased after *SNHG15* depletion (Figure 5B). Protein expression of *ADAM19* and *TGFBI* upon respective treatment showed the same trend in alteration (Figure 5C). Thus, these results suggested a ceRNA-regulatory mechanism among these genes.

### Functional Analysis of Genes in the ceRNA Network

**FIGURE 6:**
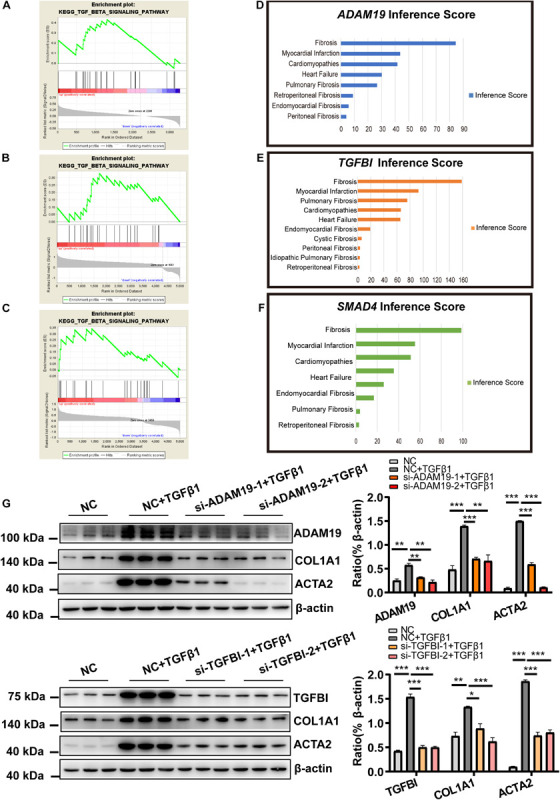
Functional analysis of mRNA in the ceRNA network (*ADAM19* and *TGFBI*). **(A)** Enriched TGF-β1 signaling pathway for *TGFBI* in the GSE116250 dataset. **(B)** Enriched TGF-β1 signaling pathway for *TGFBI* in the GSE97358 dataset. **(C)** Enriched TGF-β1 signaling pathway for *ADAM19* in the GSE116250 dataset. **(D)** Inference Score for *ADAM19*. **(E)** Inference Score for *TGFBI*. **(F)** Inference Score for *SMAD4*. **(G)** Knockdown of expression of *ADAM19* and *TGFBI* reduced expression of myofibroblast-associated genes. *n* = 3 per group. *P < 0.05, **P < 0.01, ***P < 0.001.

We carried out SGSEA to explore the relationships between gene sets according to the two mRNA in the ceRNA network (*ADAM19* and *TGFBI*) and classical cardiac-fibrosis pathways. The main results of SGSEA are presented in Figure 6. In brief, nearly all the analyses, no matter mRNA used (*ADAM19* or *TGFBI*) in gene sets division or samples the dataset derived from (ventricles or cells), yielded the TGF-β1 pathway, which is the most canonical pathway during cardiac fibrosis (Figures 6A–C). Moreover, the cell-cycle pathway (which is crucial for cell proliferation) and the interaction between cytokines and cytokine receptors (which is crucial for cytokine function) were involved (Supplementary Figure 6).

The functions of the genes in the ceRNA network were also explored by CTD. Results supported the important role of *ADAM19* and *TGFBI* as revealed by the analyses shown above, with an Inference Score of 84 and 155, respectively (Figures 6D–F). These Inference Scores showed that *ADAM19* and *TGFBI* were closely related to fibrosis, which were similar (*ADAM19*, 84.39) and much closer (*TGFBI*, 158.86) than the relationship between SMAD Family Member 4 (*SMAD4*) and fibrosis (98.75).

The analyses shown above strongly implied the role of *ADAM19* and *TGFBI* in fibrosis. Therefore, next we explored their role during HCF activation. HCF were treated by TGF-β1 with siRNA against *ADAM19* (or *TGFBI*) or NC, and expression of *COL1A1* and *ACTA2* determined. Knockdown of expression of *ADAM19* or *TGFBI* attenuated the increased expression of *COL1A1* and *ACTA2* caused by TGF-β1 (Figure 6G).

**FIGURE 7:**
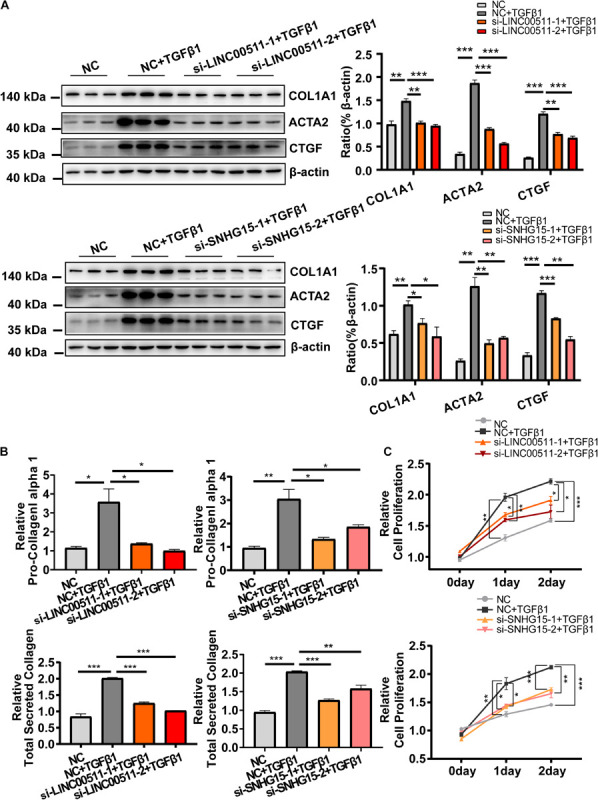
Functional validation of *LINC00511* and *SNHG15* in activation of cardiac fibroblasts. **(A)** Knockdown of expression of *LINC00511* and *SNHG15* reduced expression of myofibroblast-associated genes. **(B)** Depletion of *LINC00511* and *SNHG15* can reduce collagen secretion in HCF-cultured supernatants. **(C)** Depletion of *LINC00511* and *SNHG15* can reduce myofibroblast proliferation in 1 and 2 days, respectively. *n* = 3 per group. *P < 0.05, **P < 0.01, ***P < 0.001.

We continued to explore the role of *LINC00511* and *SNHG15* in HCF activation. Similar to the protocol mentioned above, expression of *LINC00511* and *SNHG15* was suppressed by transfection of siRNA oligonucleotides, and markers of HCF activation evaluated. As expected, we found that inhibition of expression of *LINC00511* or *SNHG15* significantly reduced expression of *COL1A1* and *ACTA2*, and collagen production (Figures 7A,B). Moreover, down-regulation of expression of *LINC00511* or *SNHG15* reversed the proliferative potency of HCF conferred by TGF-β1 (Figure 7C).

Taken together, these results suggest that the mRNA and lncRNA in the ceRNA network are crucial in TGFβ1-induced HCF activation.

## Discussion

Cardiac fibrosis is a common pathophysiologic companion of cardiovascular disease and is associated with ventricular remodeling and a poor prognosis.

To explore the key mechanisms and molecules involved in cardiac fibrosis comprehensively, we used bioinformatics analysis to identify critical genes and to predict a ceRNA regulatory network. We revealed that *ADAM19* and *TGFBI* were the most important mRNA during fibrosis. We proposed that three “hub” microRNA and two “hub” lncRNA were involved in the regulation of *ADAM19* and *TGFBI* in a ceRNA-based manner.

We applied strict criteria for screening key genes in the regulatory network. First, DE-mRNA in the regulatory network were derived from *in vivo* and *in vitro* data. DE-mRNA was identified, respectively, in fibrotic ventricles and fibrotic-induced CF. DE-mRNA derived from the ventricles typically represented *in vivo* biologic alterations during fibrosis and cardiac remodeling (but specific cell types were not identified), whereas *in vitro* data provided CF-specific gene alterations. Thus, overlapping DE-mRNA from *in vivo* and *in vitro* data provided CF-specific gene alterations during cardiac remodeling based on comprehensive and rigorous screening. MicroRNA that could target these DE-mRNA involved in the regulatory network had to meet the criteria of all five popular formulae. This strategy ensured the reliability of potential microRNA in regulation of mRNA targeting. Furthermore, all the RNA in the ceRNA network were confirmed by our experimental data. This approach ensured the possibility of ceRNA regulatory mechanisms participating in CF activation. Besides, the role of screened mRNA and lncRNA in the ceRNA network was validated. These approaches provided the selection of the most plausible candidate genes and the mechanisms of their crosstalk during cardiac fibrosis.

On the basis of the ceRNA network we constructed, we identified some novel, plausible target genes for cardiac fibrosis. This approach may aid in better understanding the molecular mechanisms during cardiac fibrosis and may provide new therapeutic targets. *ADAM19*, a member of the ADAM family, is an endopeptidase-cleaving matrix protein (Ramdas et al., 2013). No scholars have reported the association between *ADAM19* and cardiac fibrosis, but some studies have revealed the potential role of *ADAM19* in renal fibrosis and pulmonary fibrosis. *ADAM19* expression is induced by TGF-β1 and other fibrotic stimuli in alveolar epithelial cells and renal cells, and abolishment of *ADAM19* expression counteracts renal fibrosis (Keating et al., 2006; Ramdas et al., 2013). Notably, *ADAM19* expression has been reported to be regulated by *miR-29*, a finding that supports the mechanism of mRNA–microRNA interaction of this gene (Ramdas et al., 2013). Furthermore, *ADAM19* is a cell-surface protein that sheds growth factors and cytokines (Qi et al., 2009). This action may explain (at least in part) the cytokine- and proliferation-related pathways enriched in our analyses.

Initially, *TGFBI* was discovered in TGFβ1-induced cells (Skonier et al., 1992), but its role in fibrosis is not known. *TGFBI* expression is induced in the injured heart, but its role in the heart is puzzling (Schwanekamp et al., 2017). Profiling of plasma proteomes has shown that *TGFBI* expression is increased in fatty livers, which has been postulated to be involved in ECM remodeling in hepatic scar-tissue formation (Schwanekamp et al., 2017). Interaction between mRNA and microRNA has also been reported to be involved in regulation of *TGFBI* expression (Yan et al., 2018).

The plausible roles of *ADAM19* and *TGFBI* in cardiac fibrosis were supported by SGSEA data, which showed the TGF-β signaling pathway to be altered significantly. The TGF-β signaling pathway is activated by the binding of TGF-β (mainly TGF-β1) to its transmembrane receptor, and formation of the ligand–receptor complex (Hata and Chen, 2016). This event causes activation of the kinase activity of the TGF-β receptor, which recruits and phosphorylates a downstream transcription factor (SMADs) and, thus, activates expression of target genes (Massagué, 2012). TGF-β signaling is essential for the repair of injured tissue, but excessive activation is also a key mechanism in cardiac fibrosis (Travers et al., 2016). Scholars have postulated that direct inhibition of the TGF-β signaling pathway may improve long-term cardiac remodeling and cardiac function (Ikeuchi et al., 2004; Edgley et al., 2012). Thus, the inhibitory effects of the TGF-β signaling pathway, by suppression of expression of *ADAM19* and *TGFBI*, may be a therapeutic option.

As for the ncRNA in the ceRNA network, since all three microRNA were reported to be involved in fibrotic diseases, we did not repeat their effects on HCF activation in the present study. *miR-9-5p* may exert a cell-specific effect during cardiac fibrosis. Inhibition of *miR-9-5p* in cardiomyocytes reverses cardiac remodeling (Xiao et al., 2019) while it suppresses CF activation (Fierro-Fernandez et al., 2015; Wang et al., 2016). Its effect on CF seems plausible because it targets *TGFBI*, which is expressed predominately in fibroblasts (Uhlen et al., 2015). There is no direct evidence showing the relationship between *miR-153-3p*, *miR-124-3p* and cardiac fibrosis, but they are involved in the fibrosis of other organs (Liang et al., 2015; Li et al., 2018; Zhou et al., 2019a,b). Therefore, we identified crucial molecules that are closely related to fibrotic processes, but knowledge of their roles in cardiac fibrosis is poor. These crucial molecules could be novel target genes if scholars focus on CF-specific mechanisms during cardiac fibrosis.

Besides mRNA and microRNA, there are two lncRNA in the ceRNA network. Currently, no data regarding the association between *LINC00511* (or *SNHG15*) and the fibrotic situation is available. In fact, no study reporting the roles of these two lncRNA on the field of cardiovascular research is available at present. But some oncological studies have shown that *LINC00511* and *SNHG15* can act as a ceRNA in promoting cell proliferation and tumorigenesis (Liu et al., 2017; Lu et al., 2018; Wu et al., 2018; Li et al., 2019, 2020). This is consistent with our finding that their depletion alleviated HCF proliferation. However, it should be noted that mouse homologous genes of the two lncRNA are still a mystery and more studies are needed.

The same attention should be paid to the results from the KEGG analysis. It showed that most genes were enriched by the pathway related to cancer. It could be reasonable since many genes are overlapped between cardiac fibrosis and cancer. For example, genes that are closely related to inflammation, collagen, and ECM deposition are both shared by them (Diakos et al., 2014; Ma et al., 2017a; Xu et al., 2019). Moreover, despite the expression data derived from cells with relatively low passage (<4) in the study (Schafer et al., 2017), the cell aging could not be completely ruled out and aging shares many common pathways to cancer (e.g., proliferation and apoptosis). Furthermore, this also reminded us that cardiac fibrosis and cancer could be co-existing in the same patient and some anti-cancer agents, such as anthracyclines and some protein receptor tyrosine kinase inhibitors (Chen et al., 2008; Frangogiannis, 2008; Jordan et al., 2016; Meléndez and Hundley, 2016), would cause or exacerbate cardiac fibrosis.

Our study had three main limitations. First, even though we validated RNA functions in the ceRNA network in HCF, their *in vivo* effects are not known. In particular, homologous lncRNA in other species have not yet been identified, rendering *in vivo* research difficult. Second, only two datasets were available, which restricted us in further running the proof of concept of the analysis pipeline. Third, despite the direct binding to the 3′-untranslated region of lncRNA and mRNA by the corresponding microRNA, that have partly been documented by studies, namely *miR-153*/*ADAM19* (Shan et al., 2015), *miR-153*/*SNHG15* (Ma et al., 2017b), *miR-9*/*TGFBI* (Schonrock et al., 2012), and *miR-124*/*LINC00511* (Huang et al., 2020; Li et al., 2019), the remaining two regulatory mechanisms were not confirmed (*miR-124*/*ADAM19* and *miR-9*/*LINC00511*) and more studies should be considered.

Despite limitations, two lncRNA, three miRNA, and two mRNA were identified as the crucial RNA transcripts during cardiac fibrosis. Based on this, we further constructed a CF-specific ceRNA regulatory network. This could help in further elucidating the molecular mechanisms underlying the cardiac fibrosis process, as well as provide plausible target genes for future studies in this field. *In vivo* studies are needed to better understand the roles of these critical genes in CF-specific mechanisms during cardiac fibrosis.

## Data Availability Statement

The datasets generated for this study can be found in the Gene Expression Omnibus (GEO) database (GSE97358 and GSE116250).

## Author Contributions

Y-XC, J-FW, and H-FZ designed the study. Q-YG, H-FZ, S-HW, Z-TC, and Z-ZW carried out data analyses. Q-YG, YX, J-TM, and Y-WL searched and screened candidate datasets and carried out statistical analyses. Q-YG and Z-TC carried out experiments *in vitro*. Q-YG, H-FZ, Z-TC, and Y-WL drafted the manuscript. Y-XC and J-FW helped to explain the critical points in the manuscript. All authors approved the final version of the manuscript.

## Conflict of Interest

The authors declare that the research was conducted in the absence of any commercial or financial relationships that could be construed as a potential conflict of interest.

## Footnotes

1 www.perl.org/
