# Supplementary material for: Construction and Analysis of a ceRNA Network in Cardiac Fibroblast During Fibrosis Based on in vivo and in vitro Data
Source: Front Genet. 2021 Jan 21;11:503256. doi: 10.3389/fgene.2020.503256 (PMC7859616; doi:10.3389/fgene.2020.503256)
Supplement: Supplementary file 1 [file Data_Sheet_1.PDF]

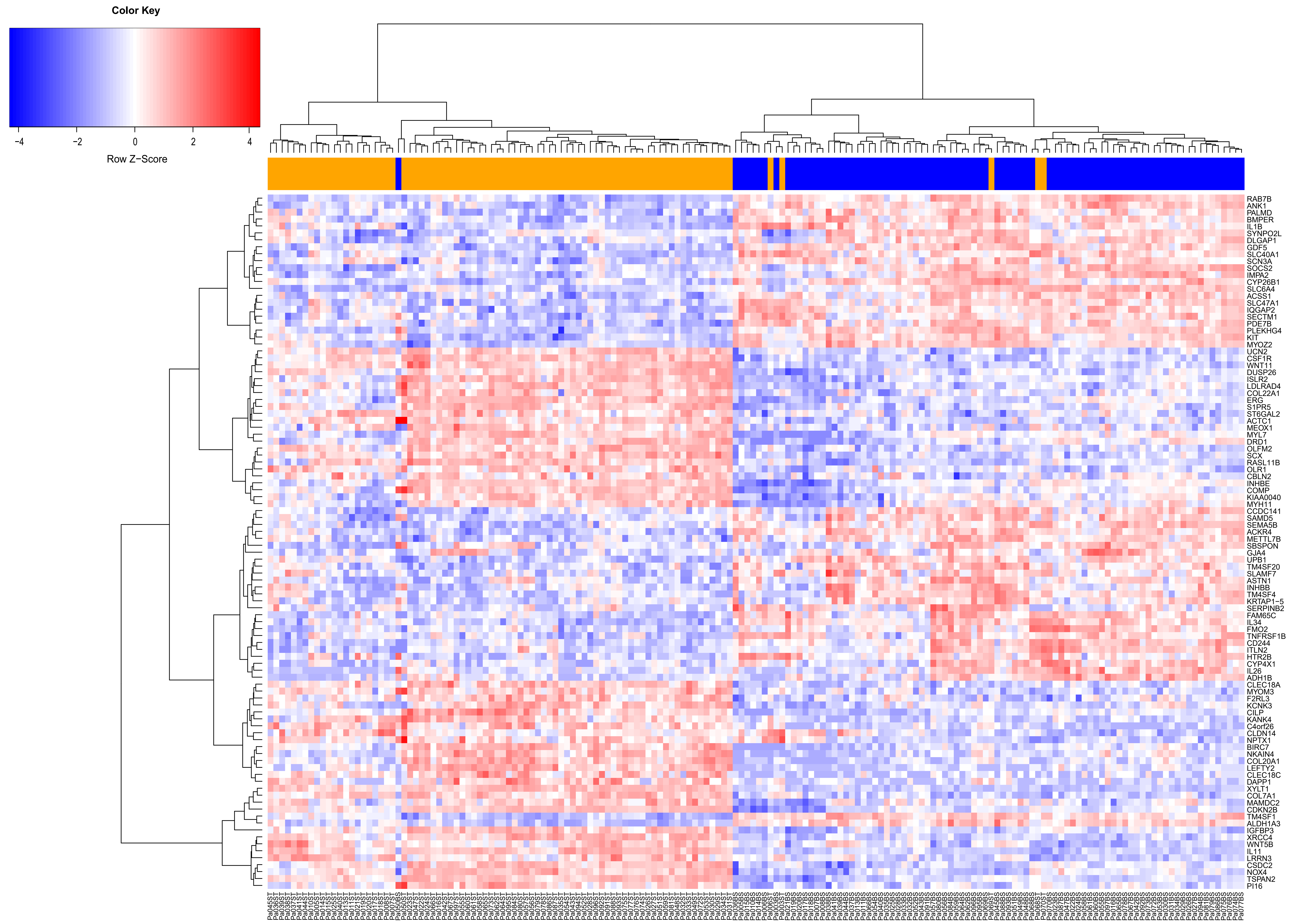
Color Key
-4
-2
0
2
4
Row Z-Score
RAB7B
ANK1
PALMD
BMPER
IL1B
SYNPO2L
DLGAP1
GDF5
SLC40A1
SCN3A
SOCS2
IMPA2
CYP26B1
SLC6A4
ACSS1
SLC47A1
IQGAP2
SECTM1
PDE7B
PLEKHG4
KIT
MYOZ2
UCN2
CSF1R
WNT11
DUSP26
ISLR2
LDLRAD4
COL22A1
ERG
S1PR5
ST6GAL2
ACTC1
MEOX1
MYL7
DRD1
OLFM2
SCX
RASL11B
OLR1
CBLN2
INHBE
COMP
KIAA0040
MYH11
CCDC141
SAMD5
SEMA5B
ACKR4
METTL7B
SBSPON
GJA4
UPB1
TM4SF20
SLAMF7
ASTN1
INHBB
TM4SF4
KRTAP1-5
SERPINB2
FAM65C
IL34
FMO2
TNFRSF1B
CD244
ITLN2
HTR2B
CYP4X1
IL26
ADH1B
CLEC18A
MYOM3
F2RL3
KCNK3
CILP
KANK4
C4orf26
CLDN14
NPTX1
BIRC7
NKAIN4
COL20A1
LEFTY2
CLEC18C
DAPP1
XYLT1
COL7A1
MAMDC2
CDKN2B
TM4SF1
ALDH1A3
IGFBP3
XRCC4
WNT5B
IL11
LRRN3
CSDC2
NOX4
TSPAN2
PI16
