# Supplementary figures and images for: Construction and Analysis of a ceRNA Network in Cardiac Fibroblast During Fibrosis Based on in vivo and in vitro Data
Source: Front Genet. 2021 Jan 21;11:503256. doi: 10.3389/fgene.2020.503256 (PMC7859616; doi:10.3389/fgene.2020.503256)

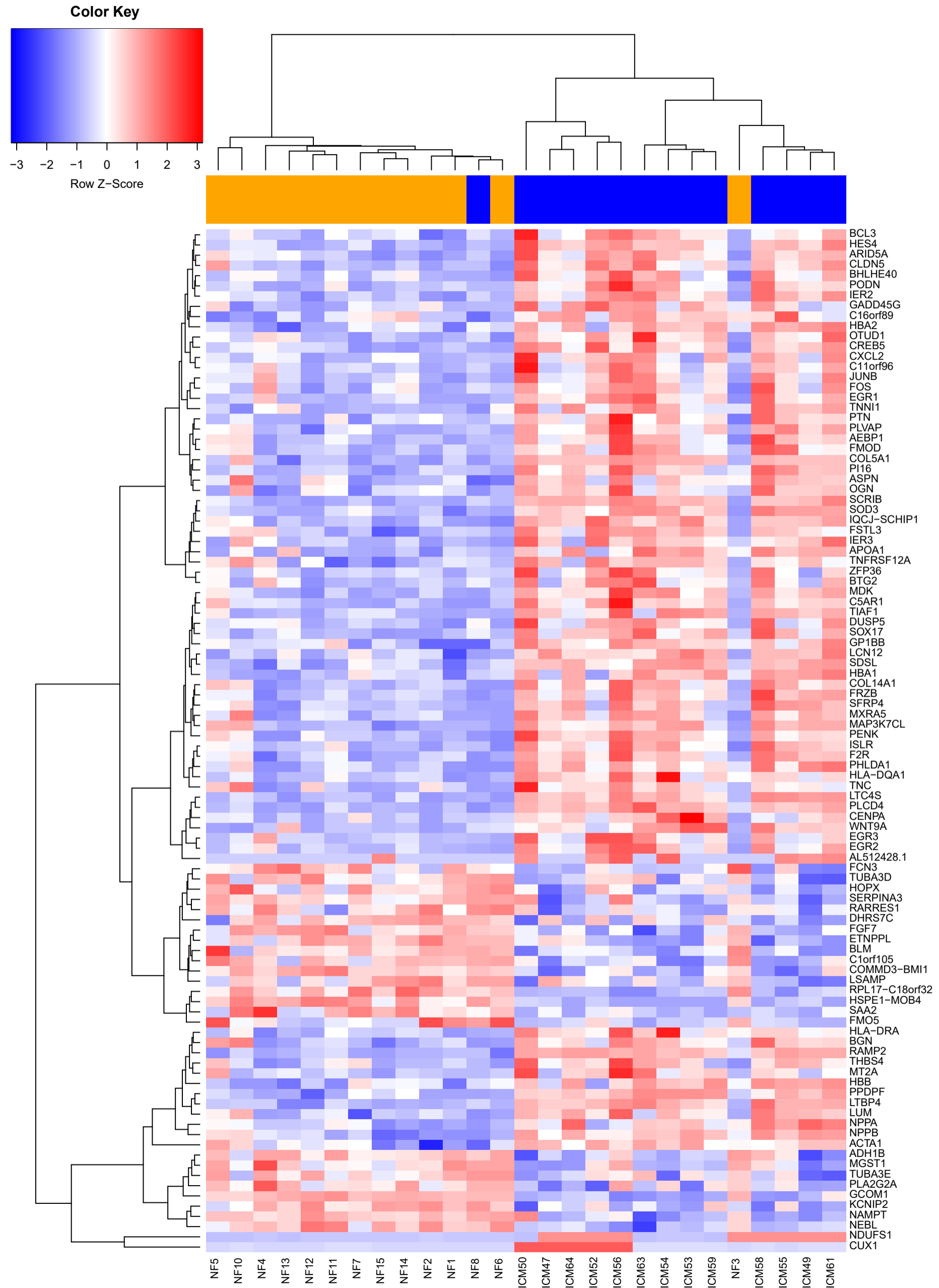

Supplement: Supplementary file 2 [file Data_Sheet_2.PDF]

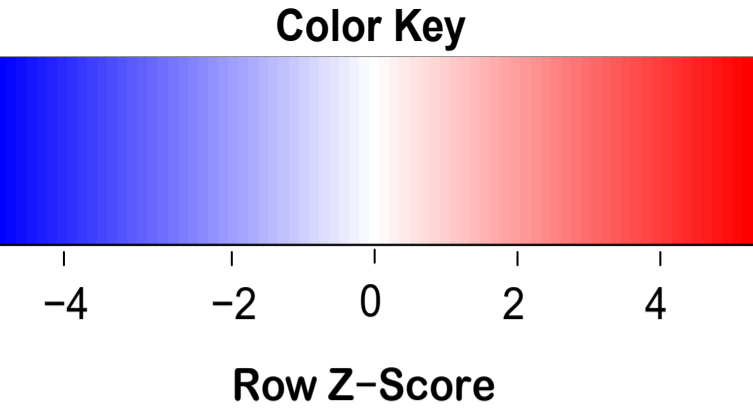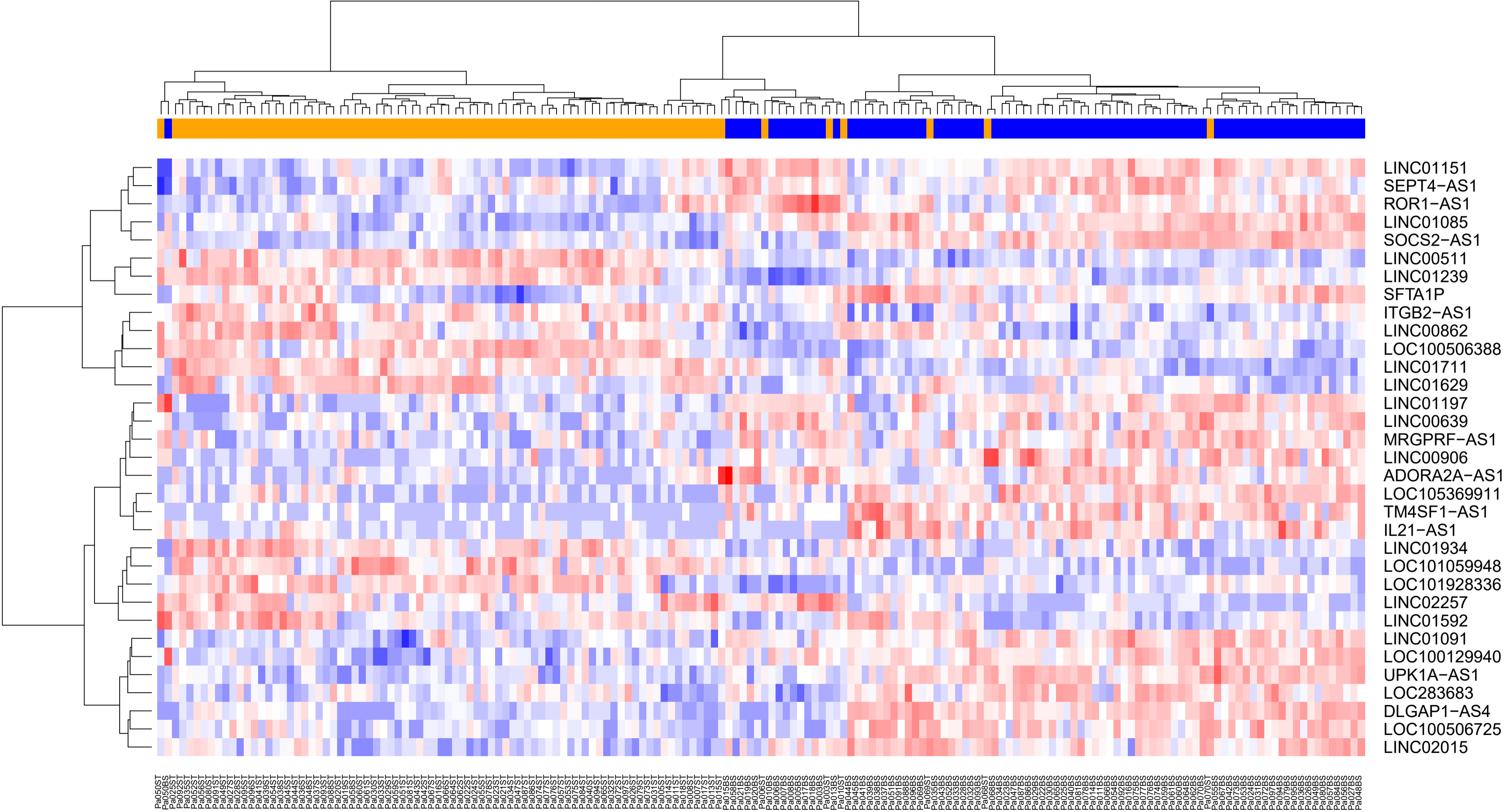

Supplement: Supplementary file 3 [file Data_Sheet_3.PDF]

Color Key

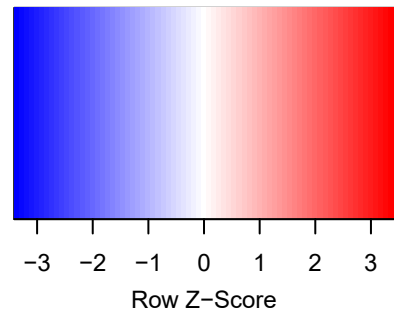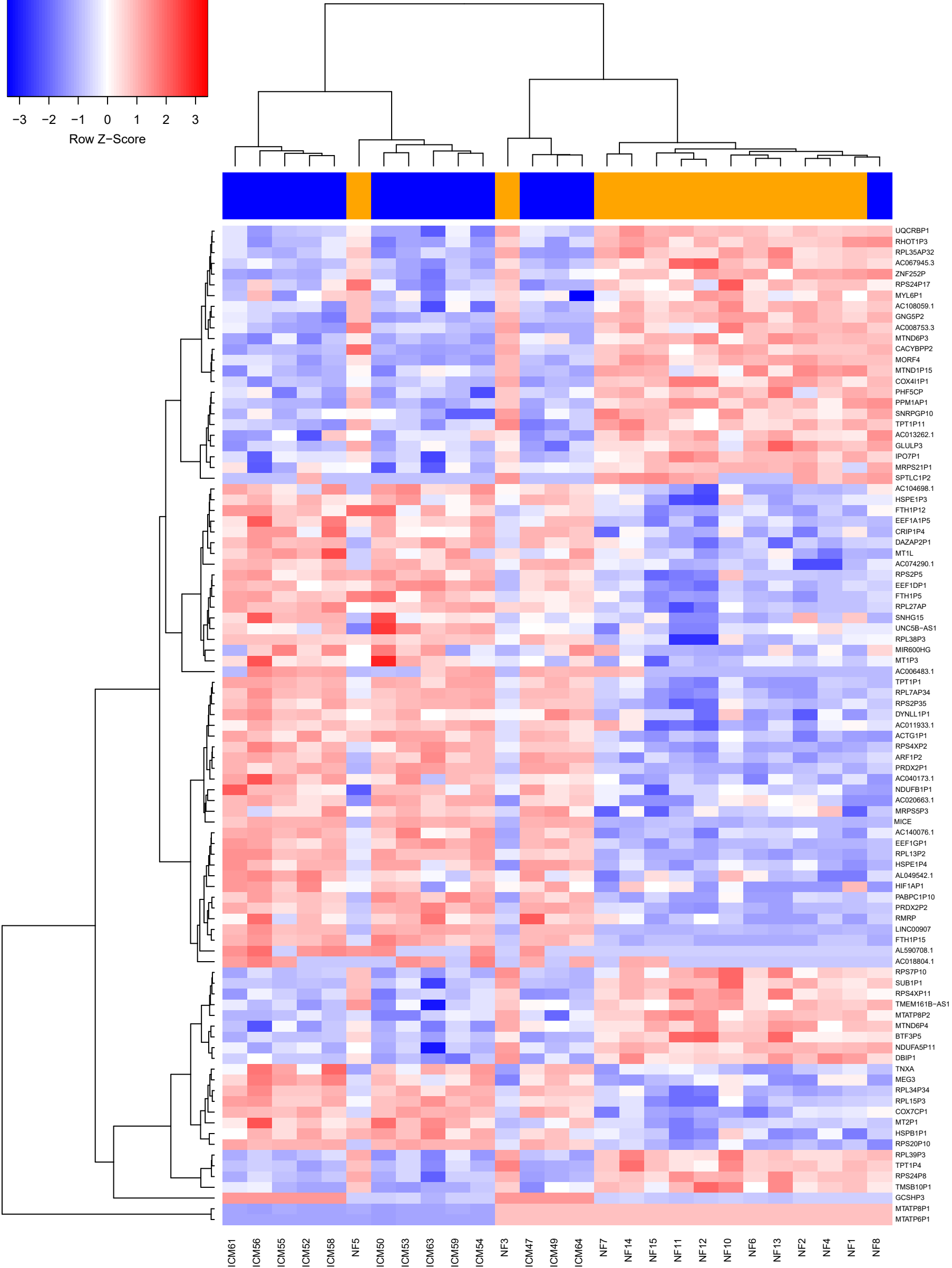

Supplement: Supplementary file 4 [file Data_Sheet_4.PDF]

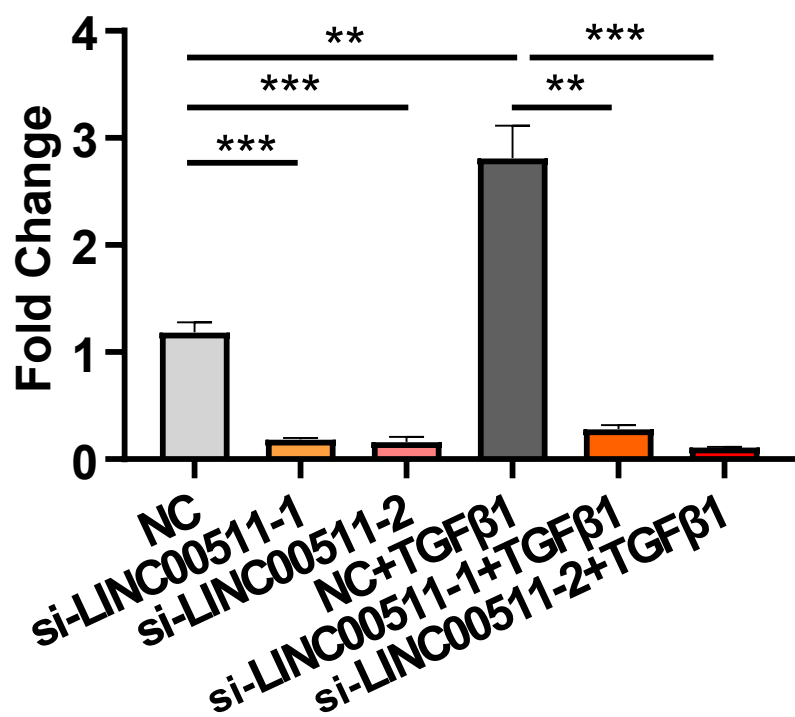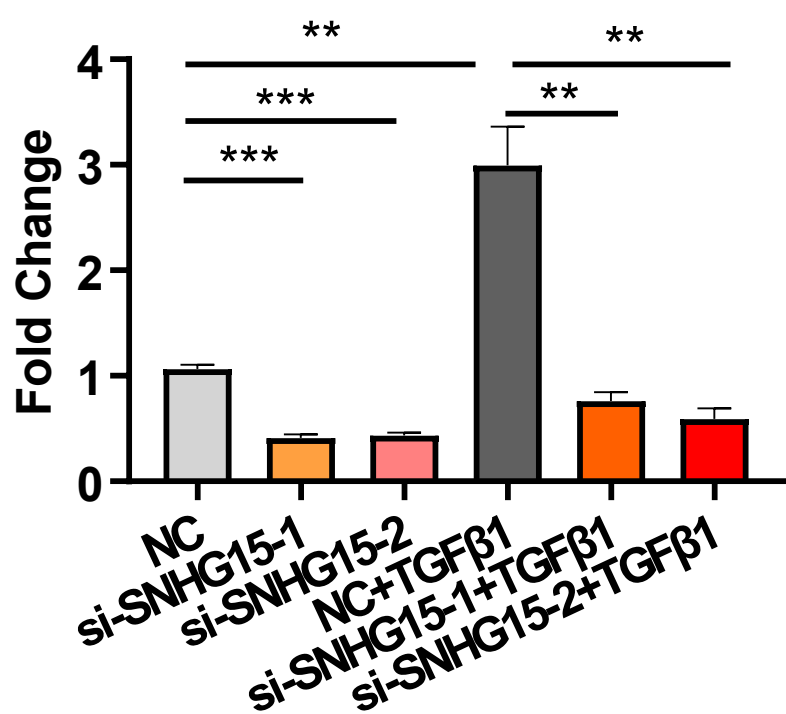

Supplement: Supplementary file 5 [file Data_Sheet_5.PDF]

A

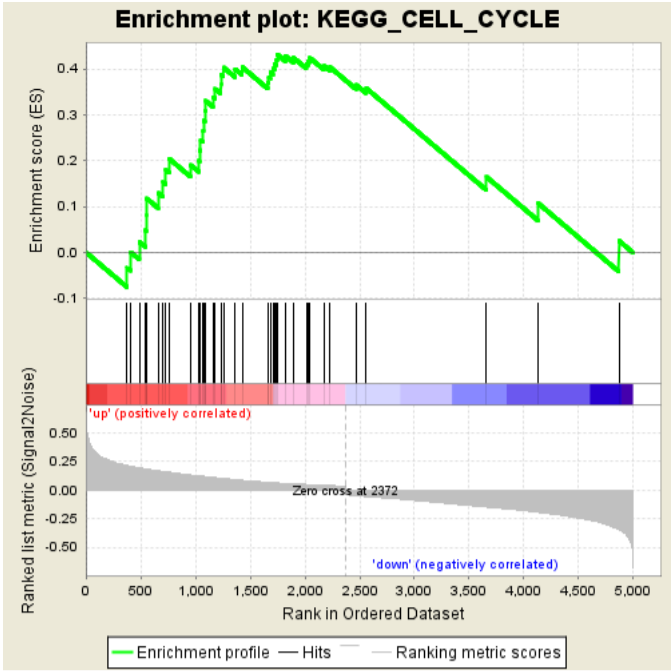

B

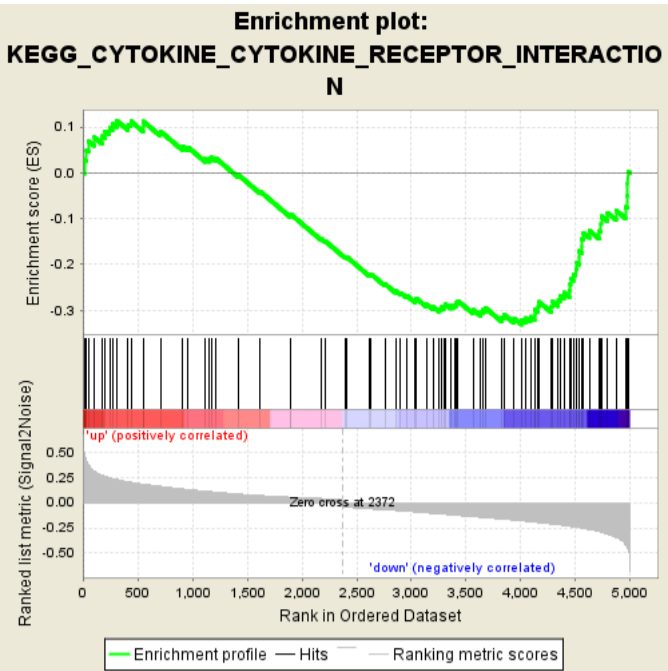

C

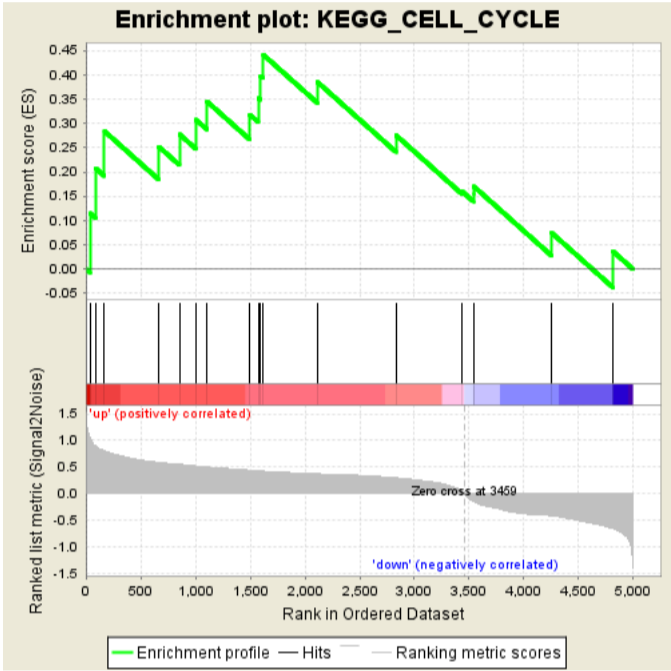

D

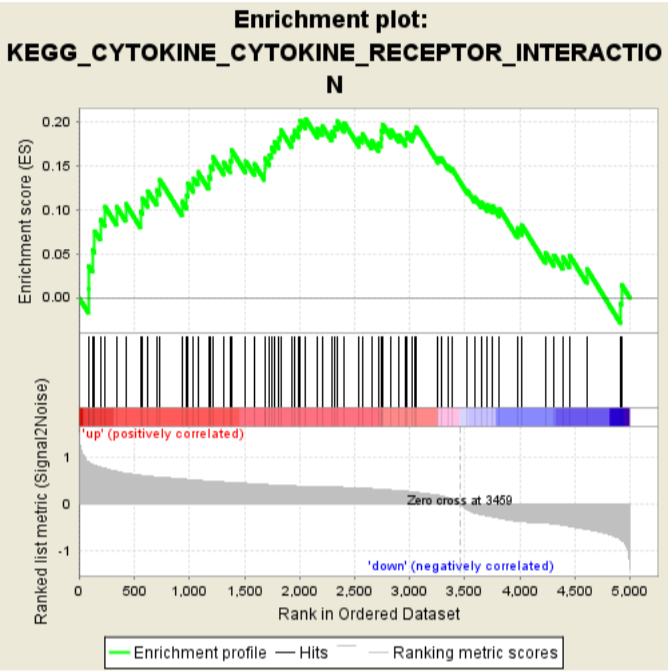

E

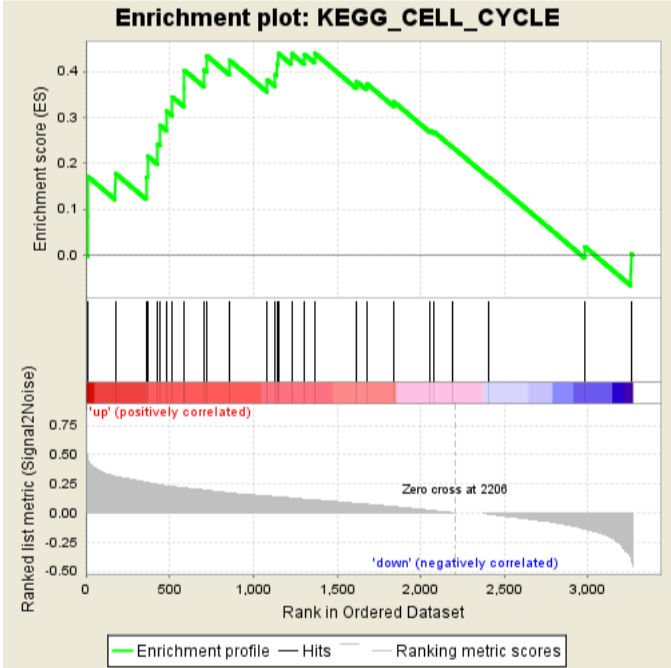

F

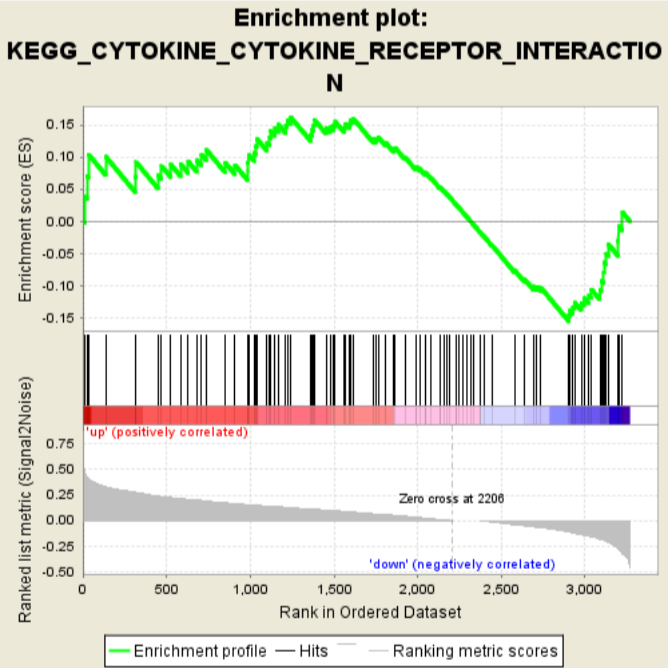

G

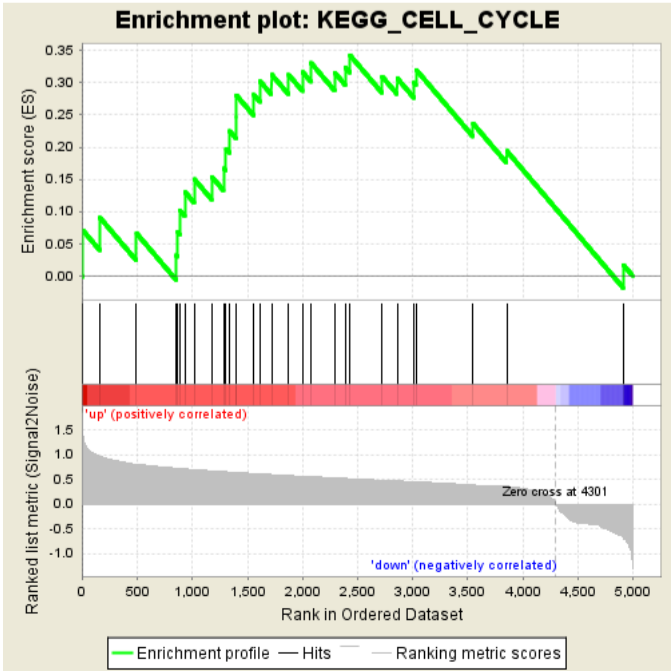

I

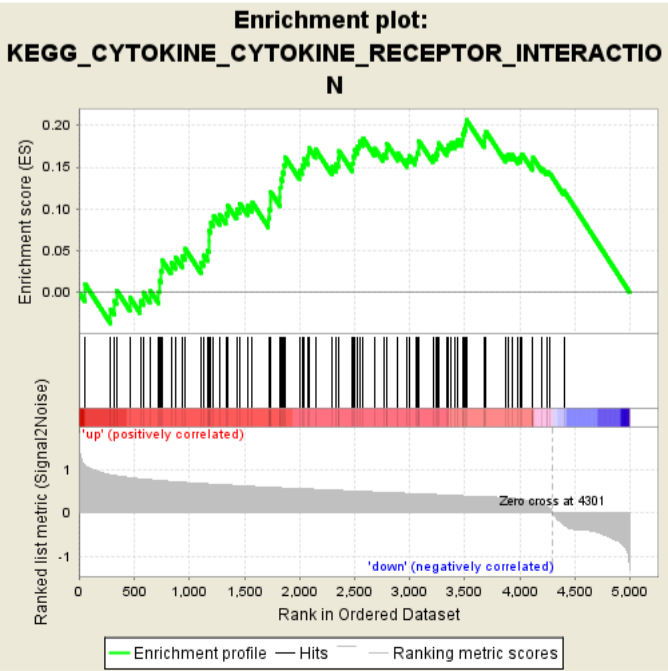

Supplement: Supplementary file 6 [file Data_Sheet_6.PDF]
