# Supplementary material for: Construction and Analysis of a ceRNA Network in Cardiac Fibroblast During Fibrosis Based on in vivo and in vitro Data
Source: Front Genet. 2021 Jan 21;11:503256. doi: 10.3389/fgene.2020.503256 (PMC7859616; doi:10.3389/fgene.2020.503256)
Supplement: Supplementary file 7 [file Presentation_1.pdf]

frontiers

Supplementary Material

Supplementary Figure 1. The heatmap for top100 DE-mRNA in GSE97358.

Supplementary Figure 2. The heatmap for top100 DE-mRNA in GSE116250.

Supplementary Figure 3. The heatmap for 39 DE-lncRNA in GSE97358.

Supplementary Figure 4. The heatmap for 93 DE-lncRNA in GSE116250.

Supplementary Figure 5. The knockdown efficiency of siRNA targeting LINC00511 and LncRNA-SNHG15 in PBS-treated HCFs and TGFβ1-treated HCFs. (A) The knockdown efficiency of siRNA-LINC00511. (B) The knockdown efficiency of siRNA-SNHG15. **, P<0.01; ***, P<0.001

Supplementary Figure 6. Supplementary results based on cardiac fibroblasts-specific DE-mRNAs in the ceRNA network (*ADAM19* and *TGFBI*). (A) ES of cell cycle for *ADAM19* in GSE97358 dataset. (B) ES of cytokine-cytokine receptor interaction for *ADAM19* in GSE97358 dataset. (C) ES of cell cycle for *ADAM19* in GSE116250 dataset. (D) ES of cytokine-cytokine receptor interaction for *ADAM19* in GSE116250 dataset. (E) ES of cell cycle for *TGFBI* in GSE97358 dataset. (F) ES of cytokine-cytokine receptor interaction for *TGFBI* in GSE97358 dataset. (G) ES of cell cycle for *TGFBI* in GSE116250 dataset. (I) ES of cytokine-cytokine receptor interaction for *TGFBI* in GSE116250 dataset.

Table S1. List of PCR primers sequence used in this study

Gene	Primer	Sequence (5'-3')
miR-25-3p	Forward	ATACATTGCACTTGTCTCG
	Reverse	CATTGCACTTGTCTCGGTCTGA
	Reverse Transcription (RT)	GTCGTATCCAGTGCGTGTCGTGGAGTCG GCAATTGCACTGGATACGACTCAGACC
miR-32-5p	Forward	CGGTATTGCACATTACTAAG
	Reverse	TATTGCACATTACTAAGTTGCA
	RT	GTCGTATCCAGTGCGTGTCGTGGAGTCG GCAATTGCACTGGATACGACTGCAACT
miR-92a-3p#	Forward	CCCTATTGCACTTGTCCCG
	Reverse	TATTGCACTTGTCCCGGCCTGT
	RT	GTCGTATCCAGTGCGTGTCGTGGAGTCG GCAATTGCACTGGATACGACACAGGCC
miR-367-3p	Forward	CCGAATTGCACTTTAGCAATG
	Reverse	AATTGCACTTTAGCAATGGTGA
	RT	GTCGTATCCAGTGCGTGTCGTGGAGTCG GCAATTGCACTGGATACGACTCACCAT
miR-26b-5p	Forward	CGGATTCAAGTAATTCAGGA
	Reverse	TTCAAGTAATTCAGGATAGGT
	RT	GTCGTATCCAGTGCGTGTCGTGGAGTCG GCAATTGCACTGGATACGACACCTATC
miR-1297	Forward	CGGTTCAAGTAATTCAGGTG
	Reverse	TTCAAGTAATTCAGGTG
	RT	GTCGTATCCAGTGCGTGTCGTGGAGTCG GCAATTGCACTGGATACGACCACCTGA

miR-30c-5p	Forward	TGTAAACATCCTACACTCTC
	Reverse	TGTAAACATCCTACACTCTCAGC
	RT	GTCGTATCCAGTGCGTGTCGTGGAGTCG<br>GCAATTGCACTGGATACGACGCTGAGA
miR-30a-5p[#]	Forward	CCTGTAAACATCCTCGACTG
	Reverse	TGTAAACATCCTCGACTGGAAG
	RT	GTCGTATCCAGTGCGTGTCGTGGAGTCG<br>GCAATTGCACTGGATACGACCTTCCAG
miR-153-3p	Forward	CCTTGCATAGTCACAAAAGT
	Reverse	TTGCATAGTCACAAAAGTGATC
	RT	GTCGTATCCAGTGCGTGTCGTGGAGTCG<br>GCAATTGCACTGGATACGACGATCACT
miR-106b-5p	Forward	CCTAAAGTGCTGACAGTGCA
	Reverse	TAAAGTGCTGACAGTGCAGAT
	RT	GTCGTATCCAGTGCGTGTCGTGGAGTCG<br>GCAATTGCACTGGATACGACATCTGCA

**Gene**	**Primer**	**Sequence (5'-3')**
miR-106a-5p[#]	Forward	GGAAAAGTGCTTACAGTGCA
	Reverse	AAAAGTGCTTACAGTGCAGGTAG
	RT	GTCGTATCCAGTGCGTGTCGTGGAGTCG<br>GCAATTGCACTGGATACGACCTACCTG
miR-874-3p	Forward	ATATCTGCCCTGGCCCGAG
	Reverse	CTGCCCTGGCCCGAGGGACCGA
	RT	GTCGTATCCAGTGCGTGTCGTGGAGTCG<br>GCAATTGCACTGGATACGACTCGGTCC
miR-590-5p	Forward	AAGAGCTTATTCATAAAAGT

	Reverse	GAGCTTATTCATAAAAGTGCAG
	RT	GTCGTATCCAGTGCGTGTCGTGGAGTCG GCAATTGCACTGGATACGACCTGCACT
miR-9-5p	Forward	TTTTCTTTGGTTATCTAGCTG
	Reverse	TCTTTGGTTATCTAGCTGTATGA
	RT	GTCGTATCCAGTGCGTGTCGTGGAGTCG GCAATTGCACTGGATACGACTCATACA
miR-26a-5p	Forward	TATTTCAAGTAAT CCAGGATA
	Reverse	TTCAAGTAATCCAGGATAGGCT
	RT	GTCGTATCCAGTGCGTGTCGTGGAGTCG GCAATTGCACTGGATACGACAGCCTAT
miR-124-3p	Forward	ATATAAGGCACGCGGTGAATG
	Reverse	TAAGGCACGCGGTGAATGCC
	RT	GTCGTATCCAGTGCGTGTCGTGGAGTCG GCAATTGCACTGGATACGACGGCATTCA
U6	Forward	CTCGCTTCGGCAGCACA
	Reverse	AACGCTTCACGAATTTGCG

Table S2. List of siRNA sequence used in this study

**Gene**	**Sequence (5'-3')**	
LINC00511-1	Forward	GGGUCGAAUUCUUCAGUUUCUTT
	Reverse	AGAAACUGAAGAAUUCGACCCTT
LINC00511-2	Forward	GCUCAAGUUCCUGACAUAAAUTT
	Reverse	AUUUAUGUCAGGAACUUGAGCTT
SNHG15-1	Forward	GCACCUUAAUUGAGCAAGUUUTT
	Reverse	AAACUUGCUCAAUUAAGGUGCTT
SNHG15-2	Forward	GACCUGACCUGAGAGAAGAUATT
	Reverse	UAUCUUCUCUCAGGUCAGGUCTT
TGFBI-1	Forward	UCCUAUUAGUGCCAAUAACCUTT
	Reverse	GUUAUUGGCACUAAUAGGAAGTT
TGFBI-2	Forward	AAGUACUUCCUAUUAGUGCCATT
	Reverse	GCACUAAUAGGAAGUACUUCATT
ADAM19-1	Forward	AAAAAGUUGCUCAUUCUUCUCTT
	Reverse	GAAGAAUGAGCAACUUUUUGCTT
ADAM19-2	Forward	AGUAUAAUGGGUUUCUGUGUATT
	Reverse	CACAGAAACCCAUUAUACUUCTT
Negative control	Forward	UUCUCCGAACGUGUCACGUTT
	Reverse	ACGUGACACGUUCGGAGAATT
